# Wear of hip prostheses increases serum IGFBP-1 levels in patients with aseptic loosening

**DOI:** 10.1038/s41598-020-79813-x

**Published:** 2021-01-12

**Authors:** Gema Vallés, Eduardo García-Rey, Laura Saldaña, Eduardo García-Cimbrelo, Nuria Vilaboa

**Affiliations:** 1 Hospital Universitario La Paz-IdiPAZ, Paseo de la Castellana 261, 28046 Madrid, Spain; 2 CIBER de Bioingeniería, Biomateriales y Nanomedicina (CIBER-BBN), Madrid, Spain; 3 Departamento de Cirugía Ortopédica y Traumatología, Hospital Universitario La Paz-IdiPAZ, Paseo de la Castellana 261, 28046 Madrid, Spain

**Keywords:** Cell biology, Biomarkers, Diseases

## Abstract

The biological mechanisms involved in aseptic loosening include inflammation-associated and bone resorption-associated processes. Coordinated cellular actions result in biochemical imbalances with devastating consequences for the joint. Given that this condition is not known for showing systemic signs, we investigated whether circulating levels of inflammation-related proteins are altered in patients with aseptic loosening. Our study included 37 patients who underwent revision surgery due to hip osteolysis and aseptic loosening and 31 patients who underwent primary total hip arthroplasty. Using antibody arrays, we evaluated the serum levels of 320 proteins in four patients from each group. The results showed differences in insulin-like growth factor-binding protein 1 (IGFBP-1) concentrations, which we then quantified using enzyme-linked immunosorbent assay tests in all study patients. The results confirmed that serum IGFBP-1 concentrations were higher in the revision surgery patients than in the hip arthroplasty patients. In vitro studies showed that exposure of human osteoblasts to titanium particles induced an IGFBP-1 release that further increased when exposure to particles was performed in media conditioned by human M1 macrophages. These findings suggest that elevated serum IGFBP-1 levels in patients with aseptic loosening can arise from increased local IGFBP-1 production in the inflammatory environment of the periprosthetic bed.

## Introduction

Total hip arthroplasty (THA) is the most successful treatment for patients suffering from osteoarthritis (OA)^1^. Advances in surgical procedures and prosthetic replacements have notably improved joint functionality and patient activity. Nonetheless, aseptic loosening remains the most common long-term complication associated with implant failure^2^. To date, the only clinical intervention for loosening is a more complex revision surgery, with poorer functional outcomes and higher complication rates than primary arthroplasty^3^. Demographic changes and a growing demand for implantation by young and active patients have led to an exponential growth in the number of primary and revision procedures. Predictive studies have indicated that this trend will increase over the coming years^4,5^ with an estimated revision burden (ratio of revision to primary THA) of approximately 17% by 2030^6^.

The etiology of aseptic loosening is multifactorial and complex, mainly arising from the biological reactions to prosthetic debris. Wear particles induce a local foreign body and chronic inflammatory response, which favors periprosthetic bone resorption and jeopardizes the osseointegration of the implant^7,8^. Resident and recruited cells involved in bone homeostasis are stimulated by wear debris in the periprosthetic area and then switch to an activated state characterized by the production of soluble mediators, including chemokines, cytokines, growth factors, degradative enzymes and free radicals, with a highly active role in the progression of osteolysis^8–12^. Changes in the levels of some of these factors have been detected in synovial fluid and serum from patients experiencing aseptic loosening and have been proposed as markers of this condition. These factors include certain pro-inflammatory cytokines, such as interleukin (IL)-1β, IL-6 and IL-8; bone metabolism-specific molecules such as receptor activator of nuclear factor-kappa B ligand (RANKL) and osteoprotegerin and anti-inflammatory mediators such as IL-10^13–15^, among others. However, it is still unclear whether these proteins can be regarded as specific biomarkers of periprosthetic osteolysis and aseptic loosening.

In this study, we found that serum insulin-like growth factor binding protein-1 (IGFBP-1) levels are higher in patients with hip aseptic loosening than in patients who underwent primary total hip arthroplasty, and therefore we evaluated whether this protein could serve as a biomarker for this disabling joint condition. Substantial amounts of metallic particles, which may initiate a cellular response contributing to periprosthetic bone resorption, have been identified in periprosthetic membranes^16–18^. To determine whether the source of elevated circulating IGFBP-1 levels is the periprosthetic cellular environment, we investigated the secretion of the protein in cultures of human osteoblasts and macrophages treated with titanium particles, as a model of metallic wear debris, or with media conditioned by osteoblasts or macrophages exposed to these particles.

## Methods

### Study design and patients

**Figure 1:**
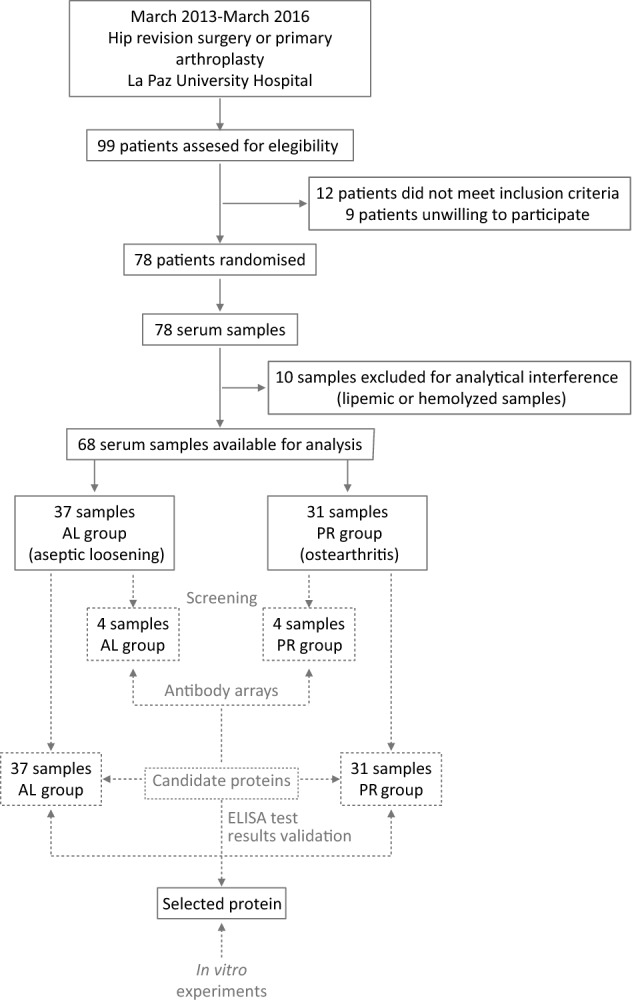
Flow diagram showing the disposition of patients and the overall study design. Continuum lines summarize the patient recruitment and origin of the serum samples. Dashed lines summarize the protocol employed for the study.

The participants enrolled in this study included patients who underwent hip revision surgery due to periprosthetic osteolysis and aseptic loosening (AL group) or primary THA (PR group). Only participants diagnosed with OA as the primary osteoarticular disease were included. The clinical and radiographic criteria reported by Altman et al*.*^19^ were used to classify the patients with hip OA. For the bilaterally operated patients, we considered only the failed hip for the classification criteria. Patients with a stable prosthesis in the contralateral joint (i.e., with no evidence or symptoms of osteolysis) were included in the AL or PR group. Only one patient in the PR group (a 70-year-old woman) had undergone previous revision surgery 2 years earlier in the contralateral joint. Aseptic loosening and the absence of sepsis were confirmed by radiological, histological, and microbiological analysis according to current diagnostic protocols^20^. Individuals undergoing immunosuppressive therapy or any other medication that could alter bone remodeling were excluded from the study. The degree and severity of the periacetabular and femoral osteolytic lesions were evaluated employing radiographic methods and classified according to the criteria of Paprosky et al*.*^21^. This study and the experimental protocols were approved by the Research Committee of Hospital Universitario La Paz (approval date: 14 February 2013). Informed consent was obtained from all participants included in the study. All protocols were conducted in accordance with the approved guidelines and regulations. The study included 68 Caucasian patients, 37 in the AL group and 31 in the PR group (see Fig. 1 for an overview of the patients and the overall study design). Table 1 shows the demographic and clinical characteristics of each group. The AL group consisted of 14 men and 23 women with a mean age of 72.1 years and a mean time since implantation of 10.4 ± 6.1 years. There were no significant differences in the distribution between the AL and PR groups regarding sex, age, bilaterality, or side.

**Table 1:** Characteristics of patients undergoing hip revision (AL) and primary THA (PR).

Variable	AL	PR	*p*
**Sex**			
Male	14 (37.8%)	15 (48.4%)	0.4631
Female	23 (62.2%)	16 (51.6%)	
**Age (years)**			
Mean ± SD	72.1 ± 9.7	68.0 ± 10.2	0.0568
Range	44–86	43–87	
**Bilateral hip prosthesis**			
Yes	7 (18.9%)	8 (25.8%)	0.5647
No	30 (81.1%)	23 (74.2%)	
**Side**			
Right	25 (67.6%)	20 (64.5%)	0.8030
Left	12 (32.4%)	11 (35.5%)	

Data are expressed in absolute and relative frequencies (%).

Within the AL group, 15 patients experienced loosening around both the femoral and acetabular component (40.5%), whereas the rest had only pelvic (46.0%) or femoral (13.5%) involvement (Table 2). Most (86.5%) patients exhibited acetabular bone defects, and approximately half (54.1%) presented femoral lesions. Most (93.3%) cases with acetabular damage were associated with type II and III femoral defects, according to Paprosky’s criteria. A larger portion of the acetabular bone defects corresponded to type 3B (43.8%), characterized by extensive and severe bone loss, lack of implant support and hip center migration associated with pelvic discontinuity. All patients included in the study had polyethylene on the acetabular side articulating against a metal femoral head provided by Traiber SL (Reus, Spain), DePuy Orthopaedics (Warsaw, IN, USA), Protek AG (Bern, Switzerland), Zimmer (Warsaw, IN, USA), Waldemar Link GmbH & Co. (Hamburg, Germany), Smith & Nephew Orthopaedics AG (Rotkreuz, Switzerland) and Stryker (Kalamazoo, MI, USA). Twenty-seven percent of the patients had cemented Charnley prostheses. Among the non-cemented prostheses (54.1%), the most common were Duraloc in the acetabulum (30%) and Profile in the femoral component (30%).

**Table 2:** Type of prostheses and bone defects in patients with aseptic loosening (AL).

Patient number	Supplier	Prosthetic component		Bone cement	Bone defect	
		Acetabular	Femoral		Acetabulum	Femur
1	Traiber SL	FlexFit	FlexFit	No	3B	IIIB
2	DePuy Orthopaedics	Charnley	Charnley	Yes	3B	IIIB
3	DePuy Orthopaedics	Charnley	Charnley	Yes	2C	IIIB
4	Protek AG	Müller	Müller	Yes	2B	
5	Protek AG	Müller	Müller	Yes	3B	
6	Zimmer	Harris-Galante	Harris-Galante	No	2B	
7	DePuy Orthopaedics	Duraloc	Profile	No	2B	
8	Waldemar Link GmbH & Co	Lubinus	Lubinus	Yes	3B	IIIB
9	Smith & Nephew Orthopaedics AG	Bicon-Plus	SL-Plus	No	3B	
10	DePuy Orthopaedics	Duraloc	Profile	No	3A	II
11	DePuy Orthopaedics	Duraloc	Profile	No	2A	II
12	DePuy Orthopaedics	Elite	Elite	Yes	3B	II
13	Traiber SL	FlexFit	FlexFit	No	2A	II
14	DePuy Orthopaedics	Charnley	Charnley	Yes	3B	
15	DePuy Orthopaedics	ACS Trilock	Profile	No	3B	
16	DePuy Orthopaedics	Duraloc	Profile	No	3B	
17	Stryker	Omnifit	Omnifit	No	3A	
18	Smith & Nephew Orthopaedics AG	Bicon-Plus	SL-Plus	No	2B	
19	Traiber SL	FlexFit	FlexFit	No	2B	IIIB
20	Zimmer	Harris-Galante	Harris-Galante	No	3B	
21	Zimmer	Harris-Galante	Harris-Galante	No	2C	
22	DePuy Orthopaedics	Charnley	Charnley	Yes	3A	IIIB
23	Zimmer	Allofit Alloclassic	Allofit Alloclassic	No		IIIA
24	Stryker	Exeter	Exeter	Yes		IIIB
25	DePuy Orthopaedics	Charnley	Charnley	Yes	3B	
26	DePuy Orthopaedics	Duraloc	Solution	No		IV
27	Stryker	Lord	Lord	No	3B	IV
28	DePuy Orthopaedics	Charnley	Charnley	Yes	2B	IIIB
29	DePuy Orthopaedics	Duraloc	Elite	Yes	2B	IIIB
30	DePuy Orthopaedics	Charnley	Charnley	Yes	3A	
31	DePuy Orthopaedics	Charnley	Charnley	Yes	3A	IIIB
32	Stryker	PCA	PCA	No	3B	
33	DePuy Orthopaedics	Profile	Profile	No	3B	
34	DePuy Orthopaedics	Charnley	Charnley	Yes	3A	
35	Protek AG	Müller	Müller	Yes		IIIB
36	DePuy Orthopaedics	Duraloc	Solution	No		IIIB
37	DePuy Orthopaedics	Charnley	Charnley	Yes	3A	IIIB

### Sample collection

Blood samples were obtained by antecubital vein puncture immediately prior to surgery, between 8 and 10 am, following an overnight fast. We collected 3 mL samples in vacutainer tubes containing a gel for serum separation and a clot activator (SST II Advance, Becton Dickinson, Plymouth, UK), which were kept for a maximum of 2 h at room temperature (RT). Serum was separated from the blood cellular components by centrifugation at 1000*g* for 10 min at RT and then stored at − 80 °C. Lipemic or hemolyzed serum samples were discarded.

### Antibody arrays

The levels of 320 proteins involved in inflammation (including cytokines, receptors and growth factors) were simultaneously analyzed in the serum samples obtained from 4 AL and 4 PR patients (age and sex-matched). In the AL group, one patient was implanted with Duraloc/Profile components (acetabular/femoral side), one was implanted with Duraloc/Solution, and two had cemented Charnley prostheses, with a mean service time of 10.4 ± 4.2 years. These 4 AL patients were representative of the total AL group in terms of prosthesis type and time of implantation. We employed a protein array based on an enzyme-linked immunosorbent assay (ELISA) system (Quantibody Human Cytokine Antibody Array 7000, QAH-CAA-7000, RayBiotech Inc., Norcross, GA, USA). Assays were performed according to the manufacturer’s instructions, using 50 µL of serum. The slides were imaged using a GenePix 4000B Microarray Scanner (Axon Instruments, Foster City, CA, USA) at 532 nm with a resolution of 5 µm per pixel. The signal densities of all spots from the arrays were processed using GenePix Pro 6.0 image analysis software (Axon Instruments). The resulting files were imported to the RayBiotech Q-Analysis Tool (QAH-CAA-7000-SW), and the data were analyzed following the manufacturer’s recommendations. Fold changes were calculated as ratios of the mean or median values of protein concentrations in the AL and PR groups. Proteins with levels differentially altered were classified and functionally categorized according to the Database for Annotation, Visualization and Integrated Discovery (DAVID; http://david.abcc.ncifcrf.gov) and the UniProt Knowledgebase (www.uniprot.org).

### Enzyme-linked immunosorbent assays

Human-specific ELISA kits were employed to measure the levels of IGFBP-1 (not the complexed form), receptor tyrosine-protein kinase erbB-2 (ErbB2), fatty acid-binding protein 2 (FABP2) and platelet endothelial cell adhesion molecule (PECAM-1) (all from R&D Systems Inc., Abingdon, UK), according the manufacturer’s instructions. The test detection limits were 6.36, 4.86, 3.63, and 21 pg/mL, respectively. The photometric measurements were performed using a Synergy 4 microplate reader (BioTek Instruments Inc., Winooski, VT, USA).

### Particles

We characterized commercially pure titanium particles (Johnson Matthey Chemicals, Ward Hill, MA, USA) in our previous studies, showing a size range considered clinically relevant (size range of 1–15 µm; 75% were < 4 µm in diameter)^18,22,23^. The titanium particles were sterilized by incubation in isopropanol at RT and dried for 48 h under UV light in a laminar flow hood^18,22–24^. The suspensions of titanium particles employed in this study had endotoxin levels of < 0.015 endotoxin units (EU)/mL, as demonstrated using the E-TOXATE assay for detection and semi-quantification of endotoxins (Sigma, Madrid, Spain). We incubated 0.1 mL of the particle suspensions (resuspended in endotoxin-free water at 5 mg/mL) with E-TOXATE reagent at 37 °C for 1 h. Endotoxin-free water and endotoxin standards derived from *Escherichia coli* were diluted at 0.015–4 EU/mL and incubated in parallel with E-TOXATE reagent. Gel formation was absent in samples that contained endotoxin levels below the detection limit of the assay.

As described in our previous studies^18,22,23^, the particles were resuspended at 20 mg/mL in growth medium, sonicated for 10 min at maximum power in a bath sonicator (Bransonic 12, Branson Ultrasonidos SAE, Barcelona, Spain) and thoroughly vortexed. Immediately after, the particle suspension was added to the cells.

### Cell culture and treatments

Primary osteoblast cultures were established from human bone specimens aseptically collected during orthopedic knee surgery and cultured as previously described^18,22–24^. Each bone sample was processed in a separated primary culture, and each experiment was performed using cells from a single patient (n = 8, mean age 79.1 ± 5.5 years). Patients enrolled in the study gave their informed consent, and the procedures using human tissue designated “surgical waste” were approved by the Human Research Committee of Hospital Universitario La Paz (approval date: 20 December 2018). Osteoblasts were cultured in growth medium consisting of Dulbecco’s modified Eagle’s medium containing 15% (v/v) fetal bovine serum (FBS), 500 IU/mL penicillin and 0.1 mg/mL streptomycin, in a humidified 5% CO_2_ atmosphere at 37 °C. The culture media were changed every 3–4 days until confluence. The osteoblastic phenotype of cells derived from bone explants was routinely confirmed by cytochemical measurement of alkaline phosphatase activity.

For the experiments, we seeded 2 × 10^5^ osteoblasts in 6-well plates, which were then cultured for 24 h, washed with phosphate buffered-saline (PBS), supplemented with 1.5 mL of growth medium and 0.5 mL of titanium particles resuspended in culture medium at 20 mg/mL (to achieve a dose of 5 mg/mL, equivalent to 1.05 mg/cm^2^), and incubated for a further 24 h. Previous reports by our group had indicated that this dose induced the osteoblastic secretion of mediators involved in bone resorption^18,23,24^. As controls, osteoblasts were incubated in the absence of particles. Conditioned media (CM) were collected from osteoblasts treated or not with titanium particles, clarified by centrifugation at 1200*g* for 10 min and stored at − 80 °C until use. To exclude cell damage due to particle exposure, the metabolic activity of osteoblasts was routinely assessed using the alamarBlue assay (Biosource, Nivelles, Belgium). Briefly, cells exposed or not to titanium particles were incubated for 3 h in growth medium containing 10% alamarBlue dye. The media were collected and the fluorescence emitted by cell-reduced alamarBlue was quantified using a Synergy 4 microplate reader (BioTek Instruments Inc.). In some of the experiments, the osteoblasts seeded in 6-well plates (as above described) were incubated for 24 h in 2 mL of a mixture of equal volumes of growth medium and CM from osteoblasts treated or not with titanium particles. At the end of the treatments, the culture media were collected, clarified by centrifugation at 1200*g* for 10 min, supplemented with a mixture of protease inhibitors (17.5 µg/mL phenylmethylsulphonyl fluoride, 1 µg/mL pepstatin A, 2 µg/mL aprotinin and 50 µg/mL bacitracin, all from Sigma) and stored at − 80 °C until further use.

Human peripheral blood mononuclear cells (PBMCs) were isolated by density gradient centrifugation from buffy coats using Ficoll-Paque Plus medium (GE Healthcare Bio-sciences, Uppsala, Sweden) as previously described^25,26^. Buffy coats were obtained from eight healthy blood donors, as anonymously provided by the Comunidad de Madrid Blood Bank (Madrid, Spain). The experimental protocols complied with the established ethical guidelines and regulations and were approved by the Human Research Committee of Hospital Universitario La Paz (approval date: 20 May 2016). For monocyte isolation, 15 × 10^6^ PBMCs were seeded in 6-well plates and allowed to adhere for 1 h in serum-free RPMI-1640 medium. Adherent cells were then cultured for 7 days in 2 mL of RPMI-1640 medium supplemented with 10% (v/v) heat-inactivated FBS, 500 UI/mL penicillin, 0.1 mg/mL streptomycin and 200 U/mL granulocyte macrophage-colony stimulating factor (GM-CSF) or 20 ng/mL macrophage-colony stimulating factor (M-CSF) (both cytokines from Peprotech, London, UK). Cytokines were added every 2 days. Macrophages generated after incubation with GM-CSF (hereafter referred to as M1 macrophages) expressed the M1-specific markers CCR7 and CD80, whereas macrophages generated after incubation with M-CSF (hereafter referred to as M2 macrophages) expressed high levels of CD163, a marker associated with the M2 phenotype^25,26^. M1 and M2 macrophages seeded in 6-well plates were washed with PBS, supplemented with 1.5 mL of growth medium and 0.5 mL of titanium particles resuspended in culture medium at 20 mg/mL (to achieve a dose of 5 mg/mL, equivalent to 1.05 mg/cm^2^), and incubated for a further 24 h. As controls, macrophages were incubated in the absence of particles. CM was collected from macrophages treated or not with titanium particles, clarified by centrifugation at 1200*g* for 10 min and stored at − 80 °C until use. The levels of tumor necrosis factor (TNF)-α in CM from macrophages were quantified using BD CBA Flex Sets (BD Biosciences, San Jose, CA, USA). The detection limit provided by the manufacturer was 3.7 pg/mL. The data were acquired using a FACSCalibur flow cytometer and analyzed with FCAP Array Software version 3.0 (BD Biosciences). There was no TNF-α detected in the media from untreated M1 or M2 macrophages. In contrast, M1 and M2 macrophages exposed to titanium particles released 30.3 ± 6.6 ng/mL and 2.5 ± 0.4 ng/mL of TNF-α, respectively. To pre-activate the macrophages, the cells were then treated with 10 ng/mL lipopolysaccharide (LPS) (Sigma) for 90 min, washed exhaustively three times with PBS and then cultured for 5 h in 2 mL of RPMI-1640 medium supplemented with 10% FBS. After pre-activation, M1 macrophages secreted higher levels of the classical pro-inflammatory cytokines TNF-α, IL-6 and IL-1β, and lower levels of IL-10 than M2 macrophages^25,26^. When required, osteoblasts were incubated for 24 h in 1 mL of CM from M1 or M2 macrophages treated or not with titanium particles and 1 mL of growth medium or in 1 mL of CM from pre-activated, LPS-treated macrophages, 0.5 mL of fresh growth medium and 0.5 mL of growth medium containing titanium particles resuspended at 20 mg/mL. At the end of the treatments, CM were collected, clarified through centrifugation at 1200*g* for 10 min, supplemented with the mixture of proteases inhibitors described above and stored at − 80 °C until further use.

The levels of IGFBP-1 secreted from the cultured cells were quantified using a human specific ELISA kit, as described above. Cells were lysed, after exhaustively washing with PBS, using a buffer containing 5 × 10^–1^ M NaCl, 5 × 10^–2^ M Tris–HCl pH 8.0 and 1% Triton X-100, and supplemented with the mixture of protease inhibitors. IGFBP-1 levels were normalized to the total protein content in cell lysates, as determined by a Bradford-based protein assay (Bio-Rad Laboratories Inc., Hercules, CA, USA), using bovine serum albumin as standard.

### Gene expression

We employed TRI Reagent (Molecular Research Center Inc., Cincinnati, OH, USA) to isolate total RNA. We also employed Transcriptor Reverse Transcriptase and an anchored-oligo (dT)_18_ primer (Roche Life Science, Indianapolis, IN, USA) to obtain complementary DNAs from total RNA. Determination of *IGFBP-1* mRNA levels was performed by real-time quantitative polymerase chain reaction using the LightCycler FastStart DNA Master SYBR Green I and a LightCycler instrument (both from Roche Life Science). *IGFBP-1* expression values were interpolated from standard curves and normalized to the mean of the expression values for glyceraldehyde 3-phosphate dehydrogenase (*GAPDH*) and beta-glucuronidase (*GUSB*) genes, which were employed as endogenous housekeeping control genes. The specific oligonucleotide primers were: *IGFBP1*, 5′-GAAGGAGCCCTGCCGAATAG-3′, (forward primer, F), 5′-CCATTCCAAGGGTAGACGCA-3′, (reverse primer, R); *GAPDH*, 5′-GTGAAGGTCGGAGTCAACG-3′ (F), 5′-GAAGATGGTGATGGGATTTCC-3′(R); and *GUSB*, 5′-AAACGATTGCAGGGTTTCAC-3′ (F), 5′-CTCTCGTCGGTGACTGTTCA-3′ (R).

### Data and statistical analysis

GraphPad Prism 6 (GraphPad, San Diego, CA, USA) and version 11.5 of the Statistical Package for the Social Sciences, (SPSS Inc., Chicago, IL, USA) were employed for the statistical analyses (mean, median, range, standard deviation, and significance of group differences) and to generate the box and whisker plots. The categorical variables were analyzed by Fisher’s test and *p* values were calculated using non-parametric Mann–Whitney U test for continuous variables. All tests were two-sided, and *p* values < 0.05 were considered significant. A receiver operating characteristic (ROC) curve analysis was employed to assess the discriminatory accuracy of protein serum levels on the basis of the area under the curve (AUC) and to determine the optimal cut-off to distinguish between AL and PR patients. The discriminatory accuracy according to the AUC was interpreted as excellent (0.9–1), good (0.8–0.89) or fair (0.7–0.79)^27^. We also calculated the sensitivity, specificity, accuracy, positive and negative likelihood, and diagnostic odds ratio. The optimal cut-off value (i.e., threshold) with the highest sensitivity and specificity was determined by estimating the minimal value of (1 − sensitivity)^2^ + (1 − specificity)^2^ and the maximum Youden’s index (*J* = sensitivity + specificity − 1) on the ROC curve^28^.

Each in vitro experiment was performed in duplicate. Data are presented as means + standard deviation (SD) of at least five independent experiments. The Shapiro–Wilk and Kolmogorov–Smirnov tests were applied to evaluate the normal distribution of all quantitative data. To analyze statistically the data, we used the Mann–Whitney U test, the one-way analysis of variance (ANOVA) test followed by the Bonferroni’s multiple comparison test or the Kruskal–Wallis test followed by the Dunn’s multiple comparison test. In all cases, the significance level was set at *p* < 0.05.

## Results

### Screening and validation of candidate proteins

**Figure 2:**
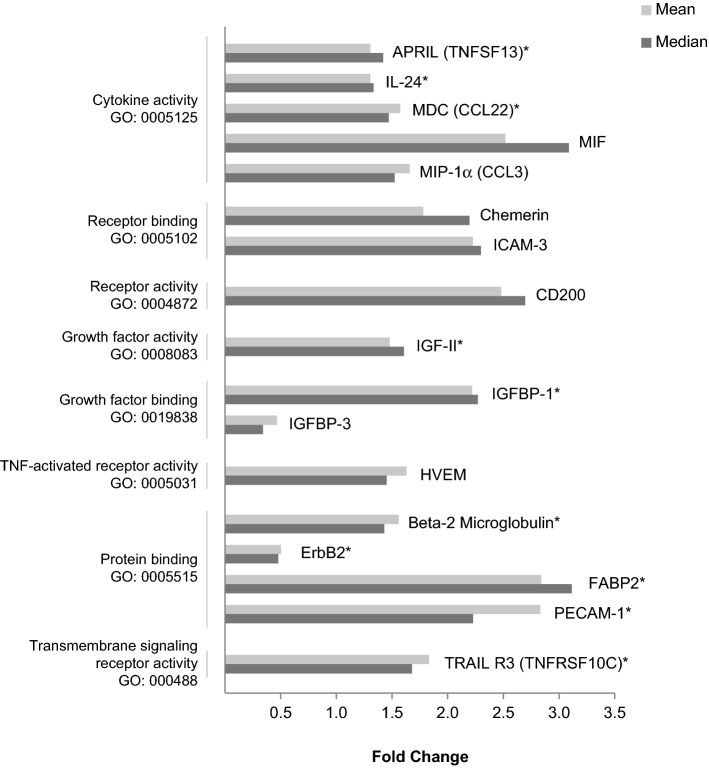
Proteins with altered serum levels in patients undergoing aseptic loosening, as quantified using antibody arrays. Bar graphs showing fold changes of mean ( ) or median ( ) values of the protein levels in the aseptic hip revision (AL) group relative to the primary THA (PR) group. Proteins were clustered according to their molecular function and gene ontology (GO) term. *p < 0.05.

**Figure 3:**
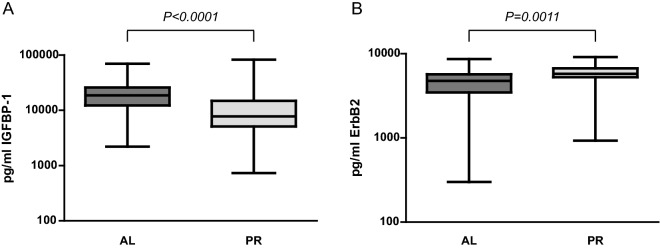
Serum IGFBP-1 (**A**) and ErbB2 (**B**) levels in patients undergoing aseptic hip revision (AL) and primary THA (PR). Results are presented as box and whisker plots in which the centerlines show the median, boxes represent the range between the first and third quartile and whiskers illustrate the highest and lowest value. *p < 0.05.

Using antibody arrays, we first quantified the levels of 320 proteins in serum samples from 4 AL patients and 4 PR patients. The concentrations of 274 proteins were within the assay’s sensitivity limits in the eight analyzed samples and were considered for further analysis. We detected that levels of 17 of the 274 proteins changed at least 1.4 fold (in either direction) between the AL and PR groups (Fig. 2). As expected, the ontology analysis revealed that most of these proteins presented cytokine, receptor or growth factor activity, and receptor or protein binding functions. We identified four differentially regulated proteins (*p* < 0.05) (Table 3), including three proteins with higher levels (median fold change from 2.23 to 3.12; IGFBP-1, FABP2 and PECAM-1) and 1 protein with lower levels (median fold change of 0.48; ErbB2) in the AL group than in the PR group. We then quantified the serum levels of IGFBP-1, FABP2, PECAM-1 and ErbB2 in the four patients from each group, employing specific ELISA assays. A good correlation was observed between the data obtained from ELISA and that from the antibody arrays. Next, we quantified the serum levels of the four proteins in 37 AL and 31 PR patients, using ELISA assays. We observed no significant differences between the AL and PR groups in the serum levels of FABP2 and PECAM-1 (data not shown). However, the median serum IGFBP-1 levels were significantly higher in the AL group than in the PR group (18,620 pg/mL vs. 7770 pg/mL, 2.40-fold, *p* < 0.0001), thereby confirming the antibody arrays data (Fig. 3A). Serum ErbB2 concentrations were significantly lower in the AL group, although the ratio with the PR group did not reach the fold-change threshold (0.82-fold, *p* = 0.0011) (Fig. 3B).

**Table 3:** Proteins differentially expressed in serum from patients undergoing aseptic hip revision (AL) and primary THA (PR), as quantified using specific-antibody arrays (in all proteins, *p* = 0.0357).

Protein	UniProtKB Accession number	Molecular function (GO) and biological process	Protein levels (pg/mL) Median value (range)	
			AL	PR
IGFBP-1	P08833	Tissue regeneration/cell growth	7978 (7762–10,429)	3512 (2733–4303)
ErbB2	P04626	Immune response/wound healing/angiogenesis	4.3 (3.6–4.5)	9.0 (5.5–9.8)
FABP2	P12104	Transport/triglyceride catabolic process/fatty acid binding	509 (333–747)	163 (119–286)
PECAM-1	P16284	Phagocytosis/Cell adhesion/ECM organization/signal transduction	2032 (1375–4253)	912 (595–1103)

### Diagnostic accuracy of serum IGFBP-1 levels

**Figure 4:**
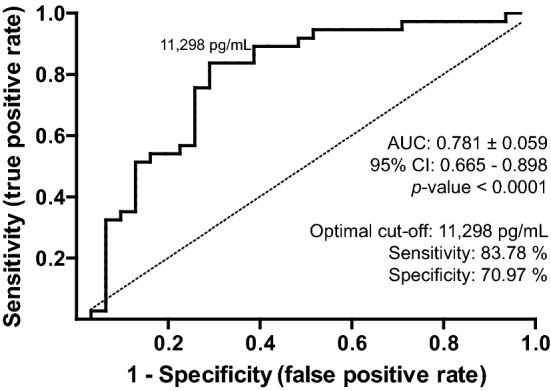
Receiver operating characteristic (ROC) curve generated for the serum IGFBP-1 levels in patients undergoing aseptic hip revision (AL) and primary THA (PR). The area under the curve (AUC) is expressed as mean ± standard error including the 95% confidence intervals (CIs). The dashed diagonal line represents the line of identity or no discrimination. The optimal cut-off value (indicated in the graph) and the associated data for sensitivity and specificity are shown.

Next, we evaluated the accuracy of serum IGFBP-1 levels in discriminating between AL and PR patients by employing an ROC analysis (Fig. 4). The AUC (0.781, *p* < 0.0001) and its respective 95% confidence interval (95% CI 0.665–0.898) indicated that the serum concentration of this protein might be useful for identifying AL patients (fair performance, AUC ≤ 0.8). The minimal value of (1 − sensitivity)^2^ + (1 − specificity)^2^ and the maximum Youden’s index in the ROC curve were 0.11 and 0.55, respectively, and the corresponding cut-off point was calculated at 11,298 pg/mL. This concentration corresponded to the highest values of sensitivity (83.78%; 95% CI 67.99–93.81) and specificity (70.97%; 95% CI 51.96–85.78). The positive and negative likelihood ratios were 2.89 and 0.23, respectively, which indicate that aseptic loosening is approximately three times more likely if IGFBP-1 levels are > 11,298 pg/mL, whereas the probability of loosening decreases to approximately 20% when the concentration is below this threshold value. Altogether, these data suggest that serum IGFBP-1 levels might help differentiate patients undergoing aseptic loosening after THA from those not affected.

### In vitro experiments

**Figure 5:**
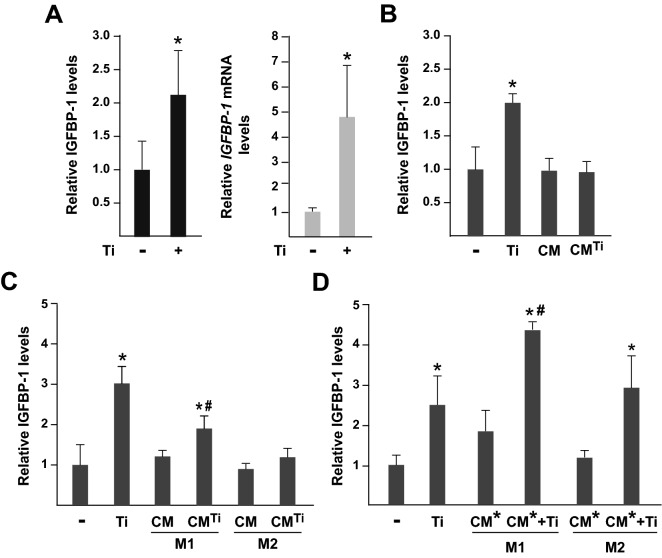
IGFBP-1 production by primary cultures of human osteoblasts. (**A**) IGFBP-1 secretion (left panel) and *IGFBP1* mRNA levels (right panel) in osteoblasts exposed (+) or not (−) to 5 mg/mL of titanium (Ti) particles for 24 h. (**B**–**D**) IGFBP-1 secretion in osteoblasts incubated for 24 h in conditioned media from osteoblasts exposed (CM^Ti^) or not (CM) to 5 mg/mL of Ti particles (**B**); in conditioned media from M1 and M2 macrophages exposed (CM^Ti^) or not (CM) to 5 mg/mL of Ti particles (**C**); and in conditioned media from pre-activated, LPS-treated M1 or M2 macrophages containing (CM* + Ti) or not (CM*) 5 mg/mL of Ti particles (**D**). Data are presented as mean + SD and expressed as relative fold induction to levels in untreated osteoblasts, which were given the arbitrary value of 1. These levels corresponded to 6.36 pg IGFBP-1 in (**A**), 6.18 pg of IGFBP-1 in (**B**), 4.99 pg in (**C**) and 5.45 pg in (**D**) per µg of total proteins. *p < 0.05 as compared with osteoblasts not exposed to particles, and ^#^*p* < 0.05 as compared with Ti-treated osteoblasts not incubated in conditioned media.

We determined whether IGFBP-1 was secreted by osteoblasts and macrophages as cell types actively involved in osteolysis induced by wear particles^8–12^. To this end, we exposed cultures of human osteoblasts and macrophages to micrometric titanium particles. Osteoblasts released IGFBP-1 into the culture media and IGFBP-1 levels increased significantly when the cells were incubated for 24 h with 5 mg/mL titanium particles (Fig. 5A, left panel). A lower dose of titanium particles (0.5 mg/mL) had no effect on IGFBP-1 secretion (data not shown). Quantification of *IGFBP1* mRNA levels in parallel cultures of titanium-treated osteoblasts revealed a fourfold increase when compared with untreated osteoblasts indicating that *IGFBP1* gene expression is positively regulated at the mRNA level by exposure to titanium particles (Fig. 5A, right panel).

Next, we evaluated whether soluble mediators released by titanium particle-treated osteoblasts modulate osteoblastic IGFBP-1 secretion. To this end, we incubated osteoblasts for 24 h with CM from osteoblasts treated or not with 5 mg/mL of titanium particles. The data in Fig. 5B showed that osteoblastic IGFBP-1 secretion was not stimulated by incubation with conditioned media from osteoblasts. IGFBP-1 levels were undetectable in the culture medium from human M1 or M2 macrophages, treated or not with 5 mg/mL of titanium particles (data not shown). Given that osteoblasts and macrophages establish paracrine interactions that contribute to the osteolytic process, we explored whether soluble factors released from M1 or M2 macrophages exposed to titanium particles modulated the IGFBP-1 secretion by osteoblasts. Exposure to CM from untreated M1 or M2 macrophages did not affect osteoblastic secretion. However, we found a significant increase in IGFBP-1 levels when osteoblasts were incubated with CM from M1 macrophages exposed to titanium, although to a lesser extent than in osteoblasts exposed to titanium (Fig. 5C). IGFBP-1 levels in the culture media of osteoblasts were not affected by incubation with CM from titanium particle-treated M2 macrophages. Lastly, we determined whether soluble factors contained in CM from pre-activated, LPS-treated macrophages could modulate the osteoblastic titanium particle-induced secretion of IGFBP-1. To this end, we incubated osteoblasts with titanium particles and CM from pre-activated M1 or M2 macrophages. The incubation of osteoblasts with titanium particles and CM from pre-activated M2 macrophages induced IGFBP-1 secretion to a similar extent as exposure to titanium particles alone (Fig. 5D). In contrast, osteoblastic titanium particle-induced secretion of IGFBP-1 further increased after incubation with CM from pre-activated M1 macrophages.

## Discussion

Previous studies have suggested that localized peri-implant bone loss might be reflected at the systemic level^29,30^. Synovial fluid analyses have provided information on biochemical changes in the joint^31^. However, the usefulness of joint aspirate as a matrix for biomarkers is a matter of debate^32^. Although a number of authors have claimed that the diagnostic accuracy and specificity of biomarkers associated with local disease are higher in synovial fluid than in the serum of AL patients^31,33^, other data indicate that the 2 parameters could be similar in these extracellular locations^34^. Lack of correspondence between observations employing serum or synovial fluid might be attributable to the different kinetics of production and turnover of the evaluated factors. Thus, proteins with short half lives and/or that are released in a restrained manner can be barely detectable at the systemic level. Nonetheless, the search for serum biomarkers associated with aseptic loosening has attracted interest, given that synovial fluid is obtained using a more invasive procedure. Research was initially focused on the serum evaluation of candidate proteins involved in bone matrix metabolism^15,35^. Given that surrogate bone markers display nonspecific variability and can be affected by several conditions, including non-skeletal diseases^36,37^, recent studies have expanded the analysis to pleiotropic molecules involved in the molecular pathogenesis of peri-implant osteolysis^15,38^. To investigate whether there is an association between aseptic loosening and circulating levels of inflammatory and growth factors, chemokines, cytokines, or their receptors, we performed a multiplexed detection of 320 proteins in the serum of AL patients, which provides a broad molecular snapshot and might uncover unidentified mediators. Antibody microarray-based technology is a precise tool for identifying the protein signature associated with biological processes, even in complex matrices such as serum. Some of the advantages over other more time-consuming proteomic techniques include not requiring sample pretreatment, low sample consumption, high specificity and sensitivity, and its multiplexing nature, thereby offering a meaningful view of the abundance of proteins simultaneously involved in specific pathways.

In vitro and in vivo studies have shown that TNF-α, IL-1β and IL-6 are pivotal mediators in the initiation and progression of local osteolysis and aseptic loosening^11,39^. Earlier studies have reported that serum TNF-α and IL-1β levels were significantly higher in AL patients compared with OA controls or patients with stable implants, whereas subsequent studies could not detect significant variations^15,40^. In accordance with the latter observations, the antibody-based arrays employed in our study detected no significant differences in the concentrations of these cytokines between the AL and PR serum samples. The main finding of our study is that serum IGFBP-1 levels are significantly higher in the AL group than in the PR group, despite overlapping ranges and significant variability, as detected by antibody-based arrays and confirmed by ELISA tests. The data analysis performed with subgroups of AL patients categorized according to the type of prosthesis, use of cement, bone defect grade, and localization (acetabulum and/or femur) revealed no significant differences for this protein (data not shown). However, we found that differences between the AL and PR groups were more significant in the patients with bone loss in the femur and acetabulum (*p* = 0.0001) than in those with involvement of only one area (*p* = 0.0045), which suggests a relationship between the extent of periprosthetic bone loss and IGFBP-1 levels. We found no correlation between the longevity or service time of the implant and IGFBP-1 levels, which could be explained by differences in the wear rate in terms of prosthetic and patient-related factors that affect the host response and thus the implant success^41,42^.

Circulating IGFBP-1, a member of the IGFBP family, binds IGFs I and II, prolonging their half-lives and altering their interaction with cell surface receptors, thereby regulating the endocrine and local actions of IGFs^43,44^. IGFBP-1 also presents local IGF-independent functions in bone cells via its RGD domain, which binds to its integrin β1 receptor on osteoclast precursors, thereby potentiating RANKL-mediated effects^45^. Interestingly, the expression of integrin β1 is up-regulated in periprosthetic membranes retrieved from patients with aseptic loosening^46^, suggesting that increased systemic IGFBP-1 levels might contribute to enhanced osteoclast activity. In fact, a positive correlation between serum IGFBP-1 levels and mineral density loss, osteoporosis and fracture risk has been observed in a cohort study with 351 postmenopausal women^47^.

Due to its IGF binding ability, IGFBP-1 is involved in glucoregulation and is regarded as a marker of insulin sensitivity^48,49^. In normal conditions, the main regulator of systemic IGFBP-1 concentrations is the fluctuation in circulating insulin levels, which inhibits the liver production of IGFBP-1^50^. Serum insulin concentrations quantified through antibody arrays showed an interindividual coefficient of variation of 29.4%, with no significant differences between the AL and PR patients. Age, diet, and lifestyle are factors associated with changes in circulating IGFBP-1 levels^48,51^. The AL and PR groups were matched by sex and age, although we cannot rule out the possible effect that dietary habits and physical activity might have had on the results of our study.

Disorders characterized by insulin resistance, such as obesity, metabolic syndrome and type 2 diabetes mellitus, are associated with a reduction in serum IGFBP-1 levels^49^. In our study, 16.22% of the AL group and 19.35% of the PR group had type 2 diabetes mellitus, while 21.62% and 41.94%, respectively, had a body mass index (BMI) ≥ 30 kg/m^2^ (Supplementary Table S1). There were no significant differences in the frequency distribution between the AL and PR groups regarding diabetes (*p* = 0.7598) or BMI (*p* = 0.1130). IGFBP-1 levels were not significantly different between patients with or without diabetes (*p* = 0.5230 for the AL group and *p* = 0.6708 for the PR group) and between those with a BMI ≥ 30 kg/m^2^ and those with a BMI < 30 kg/m^2^ (*p* = 0.6985 for the AL group and *p* = 0.2001 for the PR group). Age is another parameter related to increased circulating IGFBP-1 levels^52^. We analyzed the correlation between serum IGFBP-1 levels and age (Supplementary Fig. S1) and found a moderate, significant correlation in the PR group (r = 0.3806, *p* = 0.0347) compared with a weak and not statistically significant correlation in the AL group (r = 0.2184; *p* = 0.1940). This lack of correlation might be explained by the association between IGFBP-1 levels and the local osteolytic lesions in the aseptic loosened patients.

The sera from 6 PR patients bearing stable, non-ceramic implants in the contralateral hip showed significantly lower IGFBP-1 levels (median, 11,962 pg/mL) than the 8 age-, sex- and implant time (6.5 years)-matched AL patients (median, 18,937 pg/mL; p = 0.02). There were no statistical differences between serum levels of the PR patients bearing a stable implant and those not bearing an implant. Despite the limitations regarding the number of patients considered in this analysis, the data suggest that higher serum IGFBP-1 levels are related to aseptic loosening and not to stable fixed joint prostheses.

Lastly, an issue that remains to be elucidated is the source of the increased circulating IGFBP-1 levels in the AL patients. Although circulating IGFBP-1 mainly derives from the liver, osteoblasts also secrete IGFBP-1^53^. Using titanium particles as a model of wear debris suitable for in vitro studies^18,22,23,54,55^, we detected that active IGFBP-1 secretion from osteoblasts significantly increased after their exposure to these particles. Inflammatory mediators such as TNF-α, IL-1β and IL-6 are in vitro inducers of *IGFBP1* expression^56,57^. Previous data from our group had indicated that the expression and secretion of pro-inflammatory factors are stimulated in osteoblasts and macrophages exposed to titanium particles^18,22,23,54,55^. Therefore, local inflammatory mediators might stimulate IGFBP-1 production in the periprosthetic bone, resulting in increased serum levels in the AL patients. We observed that the incubation of osteoblasts with media conditioned by M1 macrophages challenged with titanium particles, which contain high levels of pro-inflammatory factors, stimulated the release of IGFBP-1, whereas media conditioned by osteoblasts or M2 macrophages treated with particles had no effect. Crosstalk between osteoblasts and pre-activated M1 macrophages, characterized by an active secretion of pro-inflammatory factors^25,26^, enhanced osteoblastic particle-induced IGFBP-1 secretion. According to these data, we hypothesize that increased serum IGFBP-1 levels might be a consequence of the direct exposure of osteoblasts to wear particles released from the implant to the periprosthetic space. Moreover, inflammatory factors in the microenvironment might further stimulate IGFBP-1 production by bone-forming cells exposed to particles. Future research is needed to determine whether released IGFBP-1 has an active role in aseptic loosening.

The present study identifies serum IGFBP-1 as a potential biomarker for aseptic loosening, enabling new opportunities for developing clinical diagnostic tools to guide clinical decision making and management. Further studies are needed to validate IGFBP-1 as a diagnostic and staging biomarker of disease and to explore the exact role of this protein in aseptic loosening. A clinically useful diagnostic biomarker should have a diagnostic accuracy of 90% or higher^58^. The accuracy rate for IGFBP-1 is approximately 78% in correctly classifying cases of AL in patients with hip prostheses (i.e., fair but does not achieve an excellent predictive value). The accuracy rate, sensitivity, and specificity of IGFBP-1 are similar to those reported in previous studies identifying serum osteoprotegerin and cross-linked carboxyterminal telopetide of type I collagen levels as potential biomarkers of periprosthetic osteolysis^14,35^. The fair accuracy rate of IGFBP-1 as a biomarker of aseptic loosening might be related to one of this study’s main limitations: the small number of recruited patients. Research with large cohorts and different subpopulations is required to validate the differences detected in the circulating IGFBP-1 levels. To explore whether IGFBP-1 levels increase with aseptic loosening progression, a long-term longitudinal study should be conducted. Whether variations in IGFBP-1 levels in aseptic loosening are associated with osteoarthritis needs to be assessed by including age- and sex-matched patients without osteoarthritis.

## Supplementary Information

Supplementary Information.

## Data Availability

The authors confirm that the raw/processed data supporting the findings of this study are available from the corresponding author upon reasonable request.
