# Supplementary material for: Wear of hip prostheses increases serum IGFBP-1 levels in patients with aseptic loosening
Source: Sci Rep. 2021 Jan 12;11:576. doi: 10.1038/s41598-020-79813-x (PMC7804331; doi:10.1038/s41598-020-79813-x)
Supplement: Supplementary file 1 — Supplementary Information. [file 41598_2020_79813_MOESM1_ESM.doc]

**Supplementary information**

**Wear of hip prostheses increases serum IGFBP-1 levels in patients with aseptic loosening**

Gema Vallés^1,2^, Eduardo García-Rey^2,3^, Laura Saldaña^1,2^, Eduardo García-Cimbrelo^2,3^, Nuria Vilaboa^1,2*^

^1^Hospital Universitario La Paz-IdiPAZ, Paseo de la Castellana 261, 28046 Madrid, Spain

^2^CIBER de Bioingeniería, Biomateriales y Nanomedicina (CIBER-BBN), Madrid, Spain

^3^Departamento de Cirugía Ortopédica y Traumatología, Hospital Universitario La Paz-IdiPAZ, Paseo de la Castellana 261, 28046 Madrid, Spain

^*^Corresponding author:

Nuria Vilaboa

Hospital Universitario La Paz-IdiPAZ, Paseo de la Castellana 261,

28046 Madrid, Spain.

Tel.: +34 912071034; fax: +34 917277524.

Correspondence and requests for materials should be addressed to Nuria Vilaboa (email: [nuria.vilaboa@salud.madrid.org](mailto:nuria.vilaboa@salud.madrid.org)).

**Supplementary Table S1**. Distribution of type II diabetes mellitus and body mass index (BMI) in patients undergoing aseptic hip revision (AL) and primary total hip arthroplasty (PR). Data are expressed in absolute and relative frequencies (%).

|  |  | BMI | |  |
| --- | --- | --- | --- | --- |
| Group |  | ≥ 30 kg/m^2^ | < 30 kg/m^2^ | Total |
| AL | Diabetic | 2 | 4 | 6 |
|  |  | 5.41 % | 10.81 % | 16.22 % |
|  | Non-diabetic | 6 | 25 | 31 |
|  |  | 16.22 % | 67.57 % | 83.78 % |
|  | Total | 8 | 29 | 37 |
|  |  | 21.62 % | 78.38 % | 100 % |
| PR | Diabetic | 4 | 2 | 6 |
|  |  | 12.90 % | 6.45 % | 19.35 % |
|  | Non-diabetic | 9 | 16 | 25 |
|  |  | 29.03 % | 51.61 % | 80.65 % |
|  | Total | 13 | 18 | 31 |
|  |  | 41.94 % | 58.06 % | 100 % |


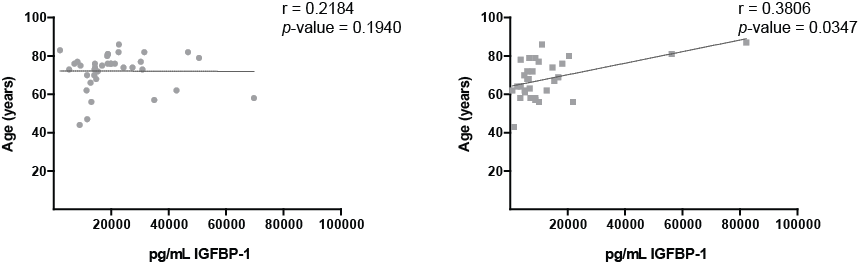


**Supplementary Figure S1.** **Correlation between serum IGFBP-1 levels and age in patients undergoing aseptic hip revision (AL,
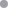
) and primary THA (PR,
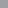
).** r represents Spearman correlation coefficient.
